# Sex-specific association of hair cortisol concentration with stress-related psychological factors in healthy young adults

**DOI:** 10.1186/s13293-021-00399-8

**Published:** 2021-10-19

**Authors:** Won Jae Kim, Kyung Mee Park, Jung Tak Park, Eunchong Seo, Suk Kyoon An, Hye Yoon Park, Eun Lee

**Affiliations:** 1 Department of Psychiatry and Institute of Behavioral Science in Medicine, Yonsei University College of Medicine, 50-1 Yonsei-ro, Seodaemun-gu, Seoul, 03722 Republic of Korea; 2 Department of Hospital Medicine, Yongin Severance Hospital, Yonsei University College of Medicine, 363 Dongbaekjukjeon-daero, Giheung-gu, Yongin, Gyeonggi-do 16995 Republic of Korea; 3 Department of Internal Medicine and Institute of Kidney Disease Research, Yonsei University College of Medicine, 50-1 Yonsei-ro, Seodaemun-gu, Seoul, 03722 Republic of Korea; 4 Department of Psychiatry, Veteran Health Service Medical Center, 53 Jinhwangdo-ro 61-gil, Gangdong-gu, Seoul, 05368 Republic of Korea

**Keywords:** Sex, Hair cortisol concentration, Stress, Emotion regulation

## Abstract

**Background:**

Hair cortisol concentration (HCC) has received attention as a useful marker of stress, but evidence on associations between psychological factors and cortisol concentration is inconsistent. The purpose of this study was to investigate the sex differences in the relationship between cortisol concentration and psychological factors in healthy young adults.

**Methods:**

A total of 205 (103 females, 102 males) healthy young adults participated. HCC and various stress-related psychological measures were compared between sexes. Multiple linear regression analyses were performed to assess associations between HCC and stress-related psychological measures for all participants and for each sex.

**Results:**

The difference in HCC according to sex was not significant. The reported number of stressful life events in the past year, stress perception, depressive and anxiety-related symptoms, and emotion dysregulation were not different between sexes, either. The association between HCC and emotion dysregulation was significant in females but not males.

**Conclusion:**

We observed a sex-specific association between HCC and psychological factors. Our findings may imply that HCC could be a useful biomarker of stress and stress-related emotion dysregulation in healthy young women.

## Highlights

- Associations between hair cortisol level and psychological factors have been inconclusive.
- In healthy young adults, the hair cortisol concentration was not significantly different between sexes.
- The association between emotion dysregulation and hair cortisol was only found in women, but not in men.
- The role of sex should be considered in utilizing hair cortisol as a biomarker for psychological stress response.

## Introduction

Cortisol is known as a biomarker that can reflect stress, and hair cortisol concentration (HCC) measurement has gained recent attention [1]. Compared to saliva or plasma cortisol, HCC corresponds to the average concentration of cortisol in the preceding months and is not influenced by diurnal variations [1]. HCC is also less invasive than other methods. These advantages make HCC more suitable for studying chronic stress than plasma- or saliva-based measurement.

Despite numerous attempts to utilize HCC as a stress biomarker, the relationship between cortisol concentration and psychological factors remains unclear [2]. This could be due to other possible moderating factors such as sex. Several sex-related differences regarding cortisol have been reported, such as higher baseline cortisol concentrations in women [3, 4]. Cortisol is the end-product of hypothalamus–pituitary–adrenal (HPA) axis responses to stress. It is downregulated by inhibiting glucocorticoid receptors in the hypothalamus and pituitary or the mineralocorticoid receptors in the hippocampus [5, 6] which is called negative feedback loop. Negative feedback of cortisol was found to be slower in women than men (for review, see [7]). Studies in rodents have found that there are fewer glucocorticoid receptors in female [8] which could also explain this sex difference in negative feedback of cortisol in human. Following psychological stress-inducing tasks, elevation and normalization of cortisol concentrations were faster in men [9]. Therefore, the relationship between HCC and psychological factors may also differ between sexes, but a possible sex-dependent relationship between HCC and psychological factors remains uncovered.

Another reason for the lack of clarity in this relationship could be that various psychological measures were not comprehensively examined. Various psychological factors have been shown to contribute differently to an individual’s cortisol response; while there was no significant association between cortisol and self-reported stress perception [2], cortisol was affected by depression and anxiety [10] and emotion regulation [11].

Since psychiatric conditions affect baseline cortisol levels or cortisol response system function [10], we performed HCC analyses in mentally healthy subjects to eliminate this confounding effect. In addition, limiting the participants’ age to young adulthood of age 18 years to 30 years old would rule out confounding effects of age on HPA axis activity [12]. The present study aimed to investigate whether sex and various stress-related psychological measures are associated with HCC in healthy young individuals. We hypothesized that there would be a sex difference in HCC, even in the absence of differences in stress-related psychological factors. Furthermore, we expected that the association between HCC and stress-related psychological factors would be sex dependent.

## Methods

### Participants

A total of 205 subjects (102 males and 103 females) were recruited from November 2016 to July 2018 via posters or internet job advertisements at Severance Hospital of the Yonsei University Health System, Seoul, Korea. Participant ages ranged from 19 to 30 years. Each individual was assessed using the Structured Clinical Interview in the Diagnostic and Statistical Manual of Mental Disorders IV to rule out major psychiatric illness. Individuals were excluded if they had a history of any of the following: (a) neurological illness; (b) head trauma accompanied by loss of consciousness; (c) a medical or surgical condition requiring hospitalization; (d) discharge from a hospital in the past 6 months; (e) use of glucocorticoid medication, oral contraceptives, or hormone replacement therapy; or (f) current pregnancy or breastfeeding.

### Measures

#### Stress-related psychological measures

Multiple psychological measures were used to assess different aspects of stress. Major stressful life events over the last year were identified with The Korean version of the Social Readjustment Rating Scale (K-SRRS) [13]. For each stressful event, the participants reported its frequency in the past 12 months, and the weighted sum over all events was calculated to produce a total score. The Cronbach’s alpha measure of internal consistency for the K-SRRS was 0.717 for the present study. The Perceived Stress Scale (PSS) [14] was used to quantify participants’ thoughts or feelings after a stressful event or encounter (range 10–50). The Beck Depression Inventory (BDI) [15] and the State–Trait Anxiety Inventory (STAI) [16] were also used, although participants with major psychiatric disorders were excluded a priori. The BDI (range 0–63) and STAI (range 20–80 for each State and Trait Anxiety Inventory) scales assessed the participants’ emotional states including depression and anxiety symptoms. The Difficulties in Emotional Regulation Scale (DERS) [17] was used to investigate the participants’ emotion dysregulation, which is the ability to regulate one’s emotions. The survey inquires how the participant behaves under negative emotions, and if they understand and accept these emotional changes. Each DERS item was scored on a five-point Likert scale, with a higher score indicating trouble accepting or regulating emotions (range 36–180).

#### HCC measurement

Approximately 10 strands or 10 mg of hair were cut with scissors from the participants’ posterior vertex. The 3 cm of hair closest to the scalp was used for cortisol measurement for each strand, which represents the average cortisol level of the preceding 3 months assuming an average growth rate of 1 cm/month. The hair samples were stored in a plastic bag at room temperature for less than 12 months. The hair protein was then extracted using a Minute™ Protein extraction kit (Invent Biotechnologies, Plymouth, MN, USA). The normalization of total amount of protein in each sample was done using a Pierce™ BCA Protein Assay Kit (Thermo Fisher Scientific, Waltham, MA, USA). Enzyme-linked immunosorbent assays were performed to detect the cortisol concentration with a detection limit of 1.14 ng/mL (lower 95% confidence limit). Most previous studies have used hair weight as reference value for HCC measurements [2]; however, hair weight can be influenced by hair characteristics, such as density or diameter [18]. Therefore, we calculated the relative HCC value by dividing the cortisol concentration by hair protein concentration [19]. The final HCC value was recorded as pg hair cortisol/μg hair protein. The mean intra-assay coefficient of variation was 3.47%.

### Statistical analysis

All statistical analyses were conducted using SPSS Version 25 (IBM Corp., Armonk, NY, USA). For parametric data including age, PSS, SAI, TAI, and DERS, independent *t*-test was used for comparisons of sexes. For non-parametric data including HCC, K-SRRS, and BDI, Mann–Whitney *U*-test was used to compare between sexes. Chi-square test was used for the comparison of level of education between sexes. In order to produce a normal distribution of responses before multiple linear regression, logarithmic, fourth root, and square root transformations were applied to the HCC, K-SRRS, and BDI scores, respectively. All variables had skewness levels that were acceptable for statistical analysis (< 1.0) after transformation. Multiple linear regression models were developed to assess associations between HCC and stress-related psychological measures for all participants and for men and women separately. Interaction effects were examined between the significant independent variables by adding interaction terms to the analysis. Participants with missing data were not included in the corresponding regression analyses. The significance threshold was *p* < 0.05 for all analyses.

## Results

### Study participants

The baseline characteristics, HCC levels, and stress-related measures of study participants are shown in Table 1. There were no significant differences in age, level of education, or stress-related psychological measures between sexes. HCC was not significantly different between sexes.

**Table 1:** Participant demographic characteristics

Variable	Total (*n* = 205)	Men (*n* = 102)	Women (*n* = 103)	*p*^d^
Age, mean (SD), years	23.0 (2.6)	23.4 (2.5)	22.7 (2.6)	0.053
Education, *n*				
High school graduation	9	3	6	0.105
College education (some)	141	78	63	
College graduation	46	19	27	
Graduate school education (some)	6	2	4	
Graduate school graduation	3	0	3	
HCC, median (IQR), pg/μg	5578.2 (6093.8)	5256.3 (5985.3)	5787.8 (5963.7)	0.286
Stress-related psychological measures				
K-SRRS^a^, median (IQR)	316.5 (420.5)	298.5 (412.8)	333.5 (403.0)	0.371
PSS^b^, mean (SD)	25.6 (5.7)	25.1 (5.6)	26.0 (5.8)	0.243
BDI^c^, median (IQR)	3.0 (7.0)	3.0 (7.0)	4.0 (6.8)	0.098
SAI^b^, mean (SD)	36.6 (9.1)	36.9 (9.4)	36.3 (8.8)	0.654
TAI^b^, mean (SD)	41.0 (7.3)	41.1 (7.7)	40.9 (7.0)	0.824
DERS^b^, mean (SD)	73.3 (18.0)	72.6 (16.9)	73.9 (19.0)	0.623

^a^K-SRRS data were available for 100 men and 102 women

^b^PSS, SAI, TAI, and DERS data were available for 101 men and all women

^c^BDI data were available for all men and 102 women

^d^*p*-value was calculated by comparing sexes using independent *t*-test for age, PSS, SAI, TAI, and DERS, Mann–Whitney *U* test for HCC, K-SRRS, and BDI, and Chi-square test for level of education

*BDI* Beck Depression Inventory, *DERS* Difficulty in Emotion Regulation Scale, *HCC* hair cortisol concentration, *IQR* interquartile range, *K-SRRS* Korean version of the Social Readjustment Rating Scale, *PSS* Perceived Stress Scale, *SAI* State Anxiety Inventory, *SD* standard deviation, *TAI* Trait Anxiety Inventory

### Associations between HCC and stress-related psychological measures

For all participants, sex (*B* = 0.248, *p* = 0.039) and emotion dysregulation (*B* = 0.012, *p* = 0.006) were significantly associated with HCC in the regression model (*R*^2^ = 0.079; *p* = 0.041). The multiple linear regression results are shown in Table 2.

**Table 2:** Multiple linear regression results of HCC for all participants

Independent variable	*B*	95% confidence interval	*p*
(Constant)	7.471	[5.942, 9.000]	< 0.001
Sex	0.248	[0.013, 0.484]	0.039
Age	0.008	[− 0.038, 0.054]	0.721
K-SRRS	0.042	[− 0.040, 0.123]	0.314
PSS	− 0.024	[− 0.051, 0.003]	0.086
BDI	− 0.023	[− 0.145, 0.098]	0.704
SAI	0.017	[− 0.002, 0.037]	0.074
TAI	− 0.013	[− 0.040, 0.014]	0.353
DERS	0.012	[0.003, 0.021]	0.006

*BDI* Beck Depression Inventory, *DERS* Difficulty in Emotion Regulation Scale, *HCC* hair cortisol concentration, *K-SRRS* Korean version of the Social Readjustment Rating Scale, *PSS* Perceived Stress Scale, *SAI* State Anxiety Inventory, *TAI* Trait Anxiety Inventory

### Sex-specific association between HCC and emotion dysregulation

**Fig. 1:**
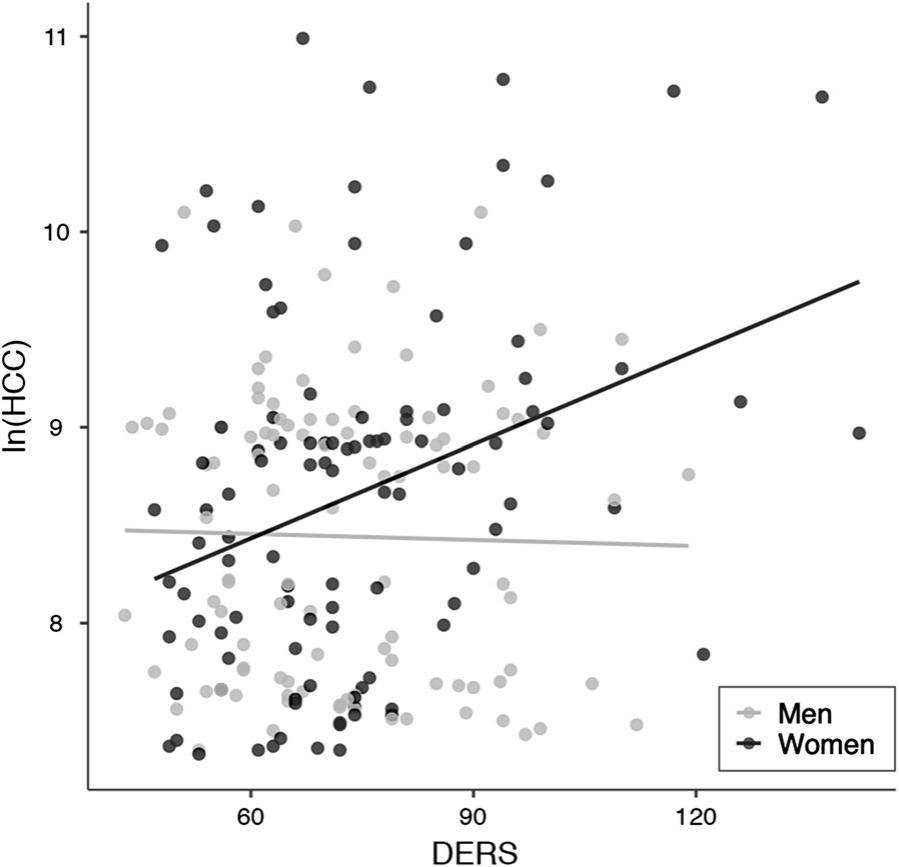
Relationships between hair cortisol concentration (HCC) and Difficulty in Emotion Regulation Scale (DERS) scores in men and women

When participants were grouped by sex, emotion dysregulation (*B* = 0.023, *p* = 0.001) was significantly associated with HCC only for females (*R*^2^ = 0.158; *p* = 0.021). None of the independent variables were significantly associated with HCC in males. The multiple linear regression results for each sex are shown in Table 3. When multiple linear regression analysis was performed with the sex*DERS interaction term, it was significantly associated with HCC (*B* = 0.017, *p* = 0.010) after adjusting for other variables. The relationships between HCC and DERS among males and females are depicted in Fig. 1.

**Table 3:** Multiple linear regression results of HCC for men and women

Sex	Independent variable	*B*	95% confidence interval	*p*
Men (*n* = 102)	(Constant)	9.107	[7.295, 10.920]	< 0.001
	Age	− 0.020	[− 0.080, 0.040]	0.514
	K-SRRS	0.014	[− 0.091, 0.120]	0.787
	PSS	− 0.027	[− 0.066, 0.013]	0.182
	BDI	0.018	[− 0.137, 0.174]	0.817
	SAI	0.020	[− 0.007, 0.046]	0.139
	TAI	− 0.007	[− 0.041, 0.026]	0.666
	DERS	− 0.001	[− 0.013, 0.011]	0.898
Women (*n* = 103)	(Constant)	6.324	[4.049, 8.599]	< 0.001
	Age	0.048	[− 0.023, 0.119]	0.182
	K-SRRS	0.080	[− 0.048, 0.209]	0.218
	PSS	− 0.019	[− 0.058, 0.020]	0.338
	BDI	− 0.076	[− 0.271, 0.118]	0.439
	SAI	0.019	[− 0.010, 0.048]	0.206
	TAI	− 0.019	[− 0.064, 0.026]	0.935
	DERS	0.023	[0.010, 0.035]	0.001

*BDI* Beck Depression Inventory, *DERS* Difficulty in Emotion Regulation scale, *HCC* hair cortisol concentration, *K-SRRS* Korean version of the Social Readjustment Rating Scale, *PSS* Perceived Stress Scale, *SAI* State Anxiety Inventory, *TAI* Trait Anxiety Inventory

## Discussion

To the best of our knowledge, this is the first study to investigate sex-dependent associations between HCC and stress-related psychological factors in a cohort of healthy young adults. In the analysis including participants of both sexes, emotion dysregulation and sex were associated with HCC levels. An association between HCC and emotion dysregulation was only observed in women, suggesting that there was a sex-specific association of HCC with stress-related psychological factors and that the association between HCC and emotion dysregulation in all participants was driven by female participants.

Stress perception, emotional states, and emotion regulation were not different between males and females in this cohort. Contrary to our original hypothesis, there was not a significant difference in HCC between males and females. Consistent with our results, several previous studies [20–22] also reported no significant differences in HCC between males and females. However, according to a recent meta-analysis [2] several studies reported higher HCC in men. Because HCC increases more in aging males than females [12], the distribution of HCC should be interpreted while also considering the influence of age. Participants in our study were young adults with a mean age of 23, which is younger than the studies which reported a sex difference in HCC (mean age of 70 in Abell et al. [23] and 42 in Wells et al. [24]). Because there is limited data on HCC in younger adults, further studies may be needed to clarify the baseline characteristics of HCC in healthy young adults.

Stratified analyses regarding sex, along with the interaction effect of sex and DERS on HCC in all participants, revealed that the association between HCC and emotion dysregulation was only significant for women. Previous studies demonstrated that the inhibition of HPA axis to downregulate cortisol is faster in men [9] possibly because they have higher glucocorticoid receptor expression in the central nervous system [7, 8]. Given that an elevated cortisol level is normalized more rapidly in males [9], the cortisol response related to emotion dysregulation may not have been reflected to higher HCC in men since HCC represents chronic cortisol elevation in preceding months [1].

Regarding relationships between HCC and various psychological factors, only emotion dysregulation was associated with HCC, and this was only observed in women. A previous study of adolescents [11] found a moderating effect of emotion regulation on HCC and stress. Our findings also underscore the importance of screening for emotion dysregulation when estimating the physiological response to stress. On the other hand, subjective stress perception measured by the PSS was not associated with HCC. This is consistent with a previous report of no significant associations between HCC and self-reported stress [2]. The K-SRRS score, which represents the past year’s major stressful life events, was not associated with HCC in our analysis. In other words, the amount of stressful events or subjective perceptions of them could have less influence on the physiological response to stress than expected. Neither symptoms of anxiety nor depression were associated with HCC in our regression model. Conversely, a previous study found that exaggerated and blunted salivary cortisol responses were associated with depression and anxiety, respectively [10]. This may indicate that associations between HCC and psychological symptoms such as depression and anxiety are moderated by other unrecognized factors. Even when anxiety and depressive symptoms or stress perception are not prominent, interventions to promote emotion regulation and coping could be more beneficial in lowering physiological responses to stress.

Our study has a few limitations. First, the data were cross-sectional, which precluded the determination of causality between variables; future longitudinal studies would be beneficial. Second, detailed medical factors or anthropometric factors, such as body mass index [2], were not considered in the analysis, although participants with severe medical diseases were excluded. Our participants were healthy young adults, and the effect of those factors would be negligible; even so, including medical or anthropometric data in the analysis might eliminate potential confounding effects. Third, the exact time scales of HCC measurement and psychological factor assessments were different. For example, K-SRRS scores represent the amount of stressful events of past year, while the cortisol level represented by 3 cm of hair roughly corresponds to the average of the past 3 months. Finally, although we excluded the potential confounding effects of race, Axis 1 psychiatric disorders, and oral glucocorticoid medication, accounting for other factors that could influence HCC could enhance our understanding, such as hair treatment or washing frequency [2], exposure to ultraviolet radiation [25], and non-oral glucocorticoid medication [26].

## Perspective and significance

Among various stress-related psychological factors, emotion dysregulation was associated with HCC in healthy young adults, particularly in females. Therefore, HCC might be a useful biomarker for stress-related psychological factors in women, especially difficulty in emotion regulation. Future studies should consider the role of sex when using HCC in analyses since the baseline level and its relationship with stress-related psychological factors were different in women compared to men.

## Data Availability

The datasets used and analyzed during the current study are available from the corresponding author on reasonable request.

## References

[1] Koumantarou Malisiova E, Mourikis I, Darviri C, Nicolaides NC, Zervas IM, Papageorgiou C (2021). Hair cortisol concentrations in mental disorders: a systematic review. Physiol Behav.

[2] Stalder T, Steudte-Schmiedgen S, Alexander N, Klucken T, Vater A, Wichmann S (2017). Stress-related and basic determinants of hair cortisol in humans: a meta-analysis. Psychoneuroendocrinology.

[3] Larsson CA, Gullberg B, Råstam L, Lindblad U (2009). Salivary cortisol differs with age and sex and shows inverse associations with WHR in Swedish women: a cross-sectional study. BMC Endocr Disord.

[4] Strewe C, Moser D, Buchheim JI, Gunga HC, Stahn A, Crucian BE (2019). Sex differences in stress and immune responses during confinement in Antarctica. Biol Sex Differ.

[5] Makino S, Hashimoto K, Gold PW (2002). Multiple feedback mechanisms activating corticotropin-releasing hormone system in the brain during stress. Pharmacol Biochem Behav.

[6] De Kloet ER, Vreugdenhil E, Oitzl MS, Joëls M (1998). Brain corticosteroid receptor balance in health and disease. Endocr Rev.

[7] Kudielka BM, Kirschbaum C (2005). Sex differences in HPA axis responses to stress: a review. Biol Psychol.

[8] Bangasser DA (2013). Sex differences in stress-related receptors: “micro” differences with “macro” implications for mood and anxiety disorders. Biol Sex Differ.

[9] Stephens MA, Mahon PB, McCaul ME, Wand GS (2016). Hypothalamic-pituitary-adrenal axis response to acute psychosocial stress: effects of biological sex and circulating sex hormones. Psychoneuroendocrinology.

[10] Fiksdal A, Hanlin L, Kuras Y, Gianferante D, Chen X, Thoma MV (2019). Associations between symptoms of depression and anxiety and cortisol responses to and recovery from acute stress. Psychoneuroendocrinology.

[11] Klosowska JC, Verbeken S, Braet C, Wijnant K, Debeuf T, De Henauw S (2020). The moderating role of emotion regulation in the association between stressors with psychological and biological measures in adolescence. Psychosom Med.

[12] Dettenborn L, Tietze A, Kirschbaum C, Stalder T (2012). The assessment of cortisol in human hair: associations with sociodemographic variables and potential confounders. Stress.

[13] Hong KE, Jeong DU (1982). Construction of Korean’ social readjustment rating scale’—a methodological study. J Korean Neuropsychiatr Assoc.

[14] Cohen S, Kamarck T, Mermelstein R (1983). A global measure of perceived stress. J Health Soc Behav.

[15] Beck AT, Steer RA. BDI, Beck depression inventory: manual. San Antonio, Tex. New York: Psychological Corp.; Harcourt Brace Jovanovich; 1987.

[16] Spielberger C, Gorsuch R, Lushene R, Vagg PR, Jacobs G. Manual for the State-Trait Anxiety Inventory (Form Y1-Y2). 1983.

[17] Gratz KL, Roemer L (2004). Multidimensional assessment of emotion regulation and dysregulation: development, factor structure, and initial validation of the difficulties in emotion regulation scale. J Psychopathol Behav Assess.

[18] Loussouarn G, Lozano I, Panhard S, Collaudin C, El Rawadi C, Genain G (2016). Diversity in human hair growth, diameter, colour and shape. An in vivo study on young adults from 24 different ethnic groups observed in the five continents. Eur J Dermatol.

[19] Frisch N, Eichler A, Plank AC, Golub Y, Moll GH, Kratz O (2020). Exploring reference values for hair cortisol: hair weight versus hair protein. Ther Drug Monit.

[20] Manenschijn L, Koper JW, Lamberts SW, van Rossum EF (2011). Evaluation of a method to measure long term cortisol levels. Steroids.

[21] Raul JS, Cirimele V, Ludes B, Kintz P (2004). Detection of physiological concentrations of cortisol and cortisone in human hair. Clin Biochem.

[22] Thomson S, Koren G, Fraser LA, Rieder M, Friedman TC, Van Uum SH (2010). Hair analysis provides a historical record of cortisol levels in Cushing's syndrome. Exp Clin Endocrinol Diabetes.

[23] Abell JG, Stalder T, Ferrie JE, Shipley MJ, Kirschbaum C, Kivimäki M (2016). Assessing cortisol from hair samples in a large observational cohort: the Whitehall II study. Psychoneuroendocrinology.

[24] Wells S, Tremblay PF, Flynn A, Russell E, Kennedy J, Rehm J (2014). Associations of hair cortisol concentration with self-reported measures of stress and mental health-related factors in a pooled database of diverse community samples. Stress.

[25] Wester VL, van der Wulp NR, Koper JW, de Rijke YB, van Rossum EF (2016). Hair cortisol and cortisone are decreased by natural sunlight. Psychoneuroendocrinology.

[26] Wester VL, Noppe G, Savas M, van den Akker ELT, de Rijke YB, van Rossum EFC (2017). Hair analysis reveals subtle HPA axis suppression associated with use of local corticosteroids: the Lifelines cohort study. Psychoneuroendocrinology.

